# Numerous Items of Interest

**Published:** 1890-10

**Authors:** 


Dr. R. H. L. Bibb at Laredo.—Our distinguished collaborator and friend, Dr. R. H. L. Bibb, of Saltillo, Mexico, being worn down with the fatigues of an extensive and laborious practice, asked for and received the appointment temporarily as quarantine officer at Laredo. This is practically a rest;—an asylum for him,—where the JOURNAL hopes, he may soon recuperate his wonted and wonderful energies of mind and body.

**No Inserts.**—Attention is called to the fact that the JOURNAL has discontinued the practice of inserting advertisements between the leaves of the reading matter, which was objected to by some of its readers. It is hard to please everybody, though we try our best to do it.

### Medical News and Miscellany.

Dr. J. F. Eaves has removed from Welbourne to Millican.

Dr. R. F. Minnock has removed from Bosqueville to Waco.

Dr. W. B. McKnight, of Springtown, is taking a special course at Bellevue and will be absent till February.

Dr. A. V. Doak, of Taylor, has been elected President of the Taylor, Llano and Corpus Christi Railroad. The Doctor has made a big fortune in real estate in the growing city of Taylor.

Wanted.—Six copies of DANIEL'S TEXAS MEDICAL JOURNAL for July, 1890, (Vol. 1, No. 6). Will pay 25 cents each for them, singly or all together. Address this office.

F. E. DANIEL, M. D.

Neutralize Nicotine.—An enterprising medical man, says a London correspondent in the *Journal of American Medical Association* for August 26, has discovered a method of rendering tobacco harmless to the mouth, heart and nerves without detriment to its aroma. A piece of cotton wool, steeped in a solution of 5 to 10 per cent. of pyrogallic acid, inserted in the pipe or cigar holder will neutralize any possible ill effects of nicotine.

Kind Words.—"I find this number 'redder' and better than ever. The editorial, 'Doctors and Doctors,' is worth a year's subscription. I think I never read anything better *put together*, or more needed than that editorial; it cuts many "Smart Alecks' to the core; it is timely and fitly spoken, and is like 'apples of gold in pictures of silver.' Long may the red-back JOURNAL live to stand up bravely and fearlessly for legitimate medicine." Yours fraternally,

B. D. S.

Dr. E. J. Ward, of Waxahachie, one of the pillars of the Texas State Medical Association, and a coming president; one of the ablest and most voluminous contributors to the Association's volumes of Transactions, the JOURNAL regrets to learn, has fallen into bad health and been compelled for a season to give up both professional and literary work. He is recuperating at Corpus Christi, where we hope the mild climate and invigorating sea breezes will soon fetch him out.

---

**The catalogue** of the New York Polyclinic shows an attendance for the session of 1889-90 of 422. The following extract shows that the Faculty have resolved to exclude all but graduates of regular medical colleges from matriculating at this school:

"Practitioners who are graduates of regular medical colleges, or who, having attended one or more courses of lectures at such college, have a legal permit to practice, will be admitted."

---

**Here He is Again.**—"P. O. Valley Mills, Sept. 24, 1890. Dear Dr you Will please be So Verrey Kind as to Send me a Sample Copy of Your Med Journal if is not asking to Much if not i Should be So Verrey glad to receve one and remember the faver in the future yours varey truly, Dr. T. F. S, eclectic."

[Verbatim.—ED.]

---

**The Sneak in the Profession**, as everywhere else, is a very contemptible fellow. He is one who professes friendship to one's face and behind one's back traduces and villifies him—*on the sly*, under the seal of secrecy. He is one who cannot strike one a blow himself—*because* of professed friendship,—but he can and does (confidentially) ask others to strike. *Can* anything be more contemptible? And—yet, Cæsar was an honorable man.

---

**We are much pained** to see a bitter personal controversy going on between two of our most esteemed contemporaries in the West. Come, brethren, drop it; life is too short and each of you can so much better occupy your time, exercise your talents and can fill your sprightly journals with matter so much more interesting to your readers. Brethren should dwell in harmony; we are all working for the same glorious end—the elevation of true medicine; let us all pull together.

Dr. Shuford's Anal Speculum.—The Parisian Inventors' Academy has conferred upon Dr. Q. A. Shuford, of Tyler, Tex., the title of "Corresponding Honorary Member, with attribution of a diploma and the great gilded medal."

The Speculum is patented in the United States. Tiernan & Co. are manufacturing it and will soon place it on the market. Our readers will be duly notified of its advent for sale through the JOURNAL's advertising columns. We have heard the Speculum spoken of in terms of high commendation.

---

"Mama, make Johnnie behave heself." "What is Johnnie doing, my son?" "Why—every time I hit him on the head with the tack-hammer—he—he—hollers!"

Certain parties take it upon themselves to persecute certain other parties; to misrepresent them, and do them every species of injustice, and when the "certain other parties" *strike back*,—a great howl is raised,—and Johnnie must be made to "behave heself." See?

---

One of Reed & Carnrick's extensive factories at Goshen, N. Y., was destroyed by fire on the 10th inst. This factory was devoted wholly to the production of their Soluble Food and Lacto-Preparata and contained extensive and valuable machinery. They had considerable stock of these Foods at their New York office, and consequently there will be no delay in filling orders. The factory will be at once rebuilt three times the size of the one burned, with machinery correspondingly enlarged.—*Dietetic Gazette.*

---

Commendation.—*Editor Daniel's Texas Medical Journal*: "I have just finished reading the September number of the 'Red Back'. In 'Quackery in and out of the Profession,' verily you have 'hewed to the line;' and I thank you for and compliment you most heartily on the able manner in which you have handled the subject. 'Lay on, MacDuff.' This is just exactly what will bring about a healthy reaction—a purging out, as it were, of unworthy—though apparently gentlemanly members of the Association. In this, I'm sure, you will have the support of all good men, in and out of the profession; and while the object may not be attained during your life time, it is sure to be reached sooner or later. With kindest regards. Yours, B."

---

The State Medical Society of Arkansas, has established a journal, to be published monthly. It was done upon recommendation of the President, and is to be the official organ of the Society. It is to be conducted on the same plan as that of the Journal of the American Medical Association. The control, editorially and otherwise, is given to five trustees, as follows: P. O. Harper, J. H. Southall and J. A. Dibrell, of Little Rock; Z. Orto, Pine Bluff, and W. B. Lawrence, Batesville. The Journal is to take the place of the yearly volume of Transactions heretofore issued, and Vol. 1, No. 1, which we have received, (July 1890), contains the President's address and a number of papers read at the last meeting, May 1890. Subscription $2 a year. Managing editor, Dr. L. P. Gibson, Little Rock, the Secretary of the Society.

---

**Prize Essays.**—The Society offers a prize of $100 for the first and $50 for the second best essay, upon any subject within the domain of Medical Jurisprudence, which is open to April 1, 1891.

The award of the Clark Bell prize of 1889 has not yet been made.

The following Committee of Award has been named, who are now considering the essays:

Judge L. A. Emery, of Maine, Chairman; Dr. W. W. Godding, of Washington, D. C.; Senator C. K. Davis, of Minn.; Judge Elisha Carpenter, of Conn.; Dr. David W. Yandell, of Ky., Prof. V. C. Vaughan, of Mich.; M. Louise Thomas, of N. Y., Secretary.

Vice-Chancellor Green, of N. J., and Prof. John Elwell, of Ohio, appointed upon the Committee, have been compelled to decline by reason of pressure of public duties, and these vacancies have been filled with the names of Dr. D. R. Wallace, of Terrell, Texas, and Judge A. L. Palmer, of New Brunswick.—*Medico-Legal Journal for January Qu.*

We are much gratified to see the name of Dr. D. R. Wallace on this committee; it is a *national recognition* of his ability and a merited compliment to a most worthy physician and laborious worker in the field of medical science.

